# Wearable Sensors Incorporating Compensatory Reserve Measurement for Advancing Physiological Monitoring in Critically Injured Trauma Patients

**DOI:** 10.3390/s20226413

**Published:** 2020-11-10

**Authors:** Victor A. Convertino, Steven G. Schauer, Erik K. Weitzel, Sylvain Cardin, Mark E. Stackle, Michael J. Talley, Michael N. Sawka, Omer T. Inan

**Affiliations:** 1 Battlefield Health & Trauma Center for Human Integrative Physiology, US Army Institute of Surgical Research, JBSA Fort Sam Houston, San Antonio, TX 78234, USA; steven.g.schauer.mil@mail.mil; 2 Uniformed Services University of the Health Sciences, Bethesda, MD 20814, USA; erik.k.weitzel.mil@mail.mil; 3 Brooke Army Medical Center, JBSA Fort Sam Houston, San Antonio, TX 78234, USA; 4 59th Medical Wing, JBSA Lackland, San Antonio, TX 78236, USA; 5 Navy Medical Research Unit, JBSA Fort Sam Houston, San Antonio, TX 78234, USA; sylvain.cardin.civ@mail.mil; 6 Commander, US Army Institute of Surgical Research, JBSA Fort Sam Houston, San Antonio, TX 78234, USA; mark.e.stackle.mil@mail.mil; 7 Commanding General, US Army Medical Research and Development Command, Fort Detrick, Frederick, MD 21702, USA; michael.j.talley4.mil@mail.mil; 8 Georgia Institute of Technology, Atlanta, GA 30332, USA; michael.sawka@biosci.gatech.edu (M.N.S.); inan@gatech.edu (O.T.I.)

**Keywords:** wearable sensors, physiology, medical monitoring, vital signs, compensatory reserve

## Abstract

Vital signs historically served as the primary method to triage patients and resources for trauma and emergency care, but have failed to provide clinically-meaningful predictive information about patient clinical status. In this review, a framework is presented that focuses on potential wearable sensor technologies that can harness necessary electronic physiological signal integration with a current state-of-the-art predictive machine-learning algorithm that provides early clinical assessment of hypovolemia status to impact patient outcome. The ability to study the physiology of hemorrhage using a human model of progressive central hypovolemia led to the development of a novel machine-learning algorithm known as the compensatory reserve measurement (CRM). Greater sensitivity, specificity, and diagnostic accuracy to detect hemorrhage and onset of decompensated shock has been demonstrated by the CRM when compared to all standard vital signs and hemodynamic variables. The development of CRM revealed that continuous measurements of changes in arterial waveform features represented the most integrated signal of physiological compensation for conditions of reduced systemic oxygen delivery. In this review, detailed analysis of sensor technologies that include photoplethysmography, tonometry, ultrasound-based blood pressure, and cardiogenic vibration are identified as potential candidates for harnessing arterial waveform analog features required for real-time calculation of CRM. The integration of wearable sensors with the CRM algorithm provides a potentially powerful medical monitoring advancement to save civilian and military lives in emergency medical settings.

## 1. Introduction

Vital signs are the most rudimentary, yet frequently relied upon physiologic data used by emergency care clinicians on which they base treatment decisions. In both prehospital and emergency department settings, vital signs are used as a primary method for triaging patients and resources for both trauma and medical encounters [1]. Pulse palpation and blood pressure have been used by physicians dating back to the 18th century with the documented work of Stephen Hales [2]. Whereas anatomical imaging diagnostics have enjoyed major advancement with novel diagnostic modalities such as computed tomography and magnetic resonance imaging in the hospital, physiological monitoring available in the prehospital and emergency room settings has remained largely unchanged. Blood pressure is still measured with a sphygmomanometer with only small incremental gains in technology over the last century. In austere clinical settings where sphygmomanometry may not be readily available (e.g., military operations, wilderness medicine), patient status is assessed by gross manual measures such as palpitation for radial pulse character and mental status [3,4,5,6]. In this regard, the Special Operations Medical Association Prolonged Field Care Working Group identified a “monitor to provide hands-free vital signs data at regular intervals” as a core capability needed to meet the requirement for prolonged field care on the battlefield [7,8]. New advancements in capturing and analyzing real-time electronic signals from the body using wearable sensor signals that are integrated with advanced computer processing capabilities hold great promise for development of novel monitoring technologies. In this review, we provide evidence for the need to use a photoplethysmographic (PPG) signal as the most informative ‘vital sign’ to be captured in emergency medical care settings. We introduce a variety of currently available wearable sensor technologies that could be used to harness PPG signals for integration with a novel predictive machine-learning algorithm designed to optimize pathophysiological monitoring and early triage decision support beyond standard vital signs.

## 2. Need to Identify New Vital Sign Measurements

### 2.1. Compromised DO_2_—A Primary Clinical Challenge to Effective Medical Monitoring

Hemorrhage is the primary reason for death after major trauma in both civilian and military settings [9,10,11,12,13]. If not controlled in its early stages, hemorrhage can result in inadequate systemic oxygen delivery (DO_2_) to vital organs (e.g., brain, heart, gut) that without effective intervention can rapidly lead to organ dysfunction and tissue death [14]. Clinically, DO_2_ is indirectly assessed by measurements of standard vital signs such as blood pressure. However, improvement in blood pressure alone does not correlate with oxygen received at the tissue level as supported by the observation that crystalloid fluids can elevate systolic pressure while simultaneously worsening patient outcomes [9]. Clinicians frequently define a measurement of systolic pressure <90 mmHg as hypotension incapable of sustaining adequate DO_2_. More recent data suggest the use of this threshold may not accurately represent risk for poor clinical outcome [4,15,16]. To this end, it has been proposed that optimal assessment of patient status demands actual measurement of systemic DO_2_ [17,18,19].

### 2.2. Current Vital Sign Monitoring

One limitation of most modern monitoring systems is a bias toward capture of only standard vital signs (Table 1). Standard vital signs exhibit little change during the early stages of volume loss due to physiological compensatory responses [20,21,22,23,24,25]. Such responses (e.g., deep inspiration, tachycardia, and vasoconstriction) regulate and maintain blood pressure and tissue perfusion prior to the onset of decompensated shock during the early stages of hemorrhage, sepsis, dehydration, and other forms of central hypovolemia [26,27,28,29].

**Figure 1:**
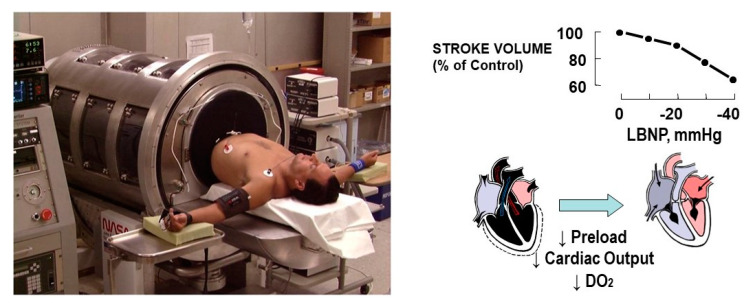
Human subject placed in the lower body negative pressure (LBNP) chamber used to induce progressive reductions in cardiac filling (preload), stroke volume, cardiac output and DO_2_ similar to hemorrhage.

In an effort to identify and compare the time course of changes in standard vital signs and physiological compensatory responses during the early stages of blood loss, lower body negative pressure (LBNP) has emerged as a validated model for controlled progressive reductions in central blood volume that mimics the physiology of hemorrhage in humans [14,31,32]. Like hemorrhage, LBNP leads to reduced filling of the heart which in turn reduces cardiac stroke volume and output, resulting in lower DO_2_ (Figure 1). Using this model of human hemorrhage has consistently revealed that commonly relied upon vital signs are not specific to the condition of blood loss or do not change until too late in the clinical course of reduced central blood volume to allow optimized patient care (Table 1).

Currently used monitors track limited vital sign measurements and chemistries on an interval basis (e.g., blood pressure) with limited capability for providing continuous, real-time physiological assessments (e.g., electrocardiogram, pulse, oxygen saturation) and are often non-specific to magnitude of hypovolemia. In addition, current commercial monitoring systems for emergency settings are bulky, power hungry, have wires interfering with patient care, and sensitive to motion artifact. Despite the above findings and limitations, clinicians continue to rely upon standard vital signs or blood chemistries when deciding to intervene because new and more effective monitoring technologies are not available.

### 2.3. Accuracy, Sensitivity, and Specificity

The effectiveness of any monitoring technology relies on its ability to provide accurate, sensitive, and specific information about the clinical condition of the patient. In this regard, it is critical that there be an assessment of the number of cases correctly identified as unhealthy (True Positive or *TP* rate), correctly identified as healthy (True Negative or *TN* rate), incorrectly identified as healthy (False Negative or *FN*), and incorrectly identified as unhealthy (False Positive or *FP*). Of course, all of this requires an agreed upon reference gold standard. Once these parameters are quantified, accuracy, sensitivity and specificity of the measurement can be assessed. Within this framework, an estimation of accuracy can be calculated as the ratio of true positive and negative cases (*TP* plus *TN*) to the sum of all measured cases. Mathematically, this can be stated as:

(1) $$\mathrm{Accuracy}=\frac{TP+TN}{TP+TN+FP+FN}$$

Since sensitivity of a measurement represents its ability to correctly identify unhealthy cases, it can be calculated as the ratio of *TP* to the sum of both true positive and false negative unhealthy cases. Mathematically, sensitivity can be stated as:

(2) $$\mathrm{Sensitivity}=\frac{TP}{TP+FN}$$

Specificity refers to the ability of a diagnostic modality to correctly identify or predict those individuals who are healthy. That is, specificity can be calculated as the ratio of *TN* to the sum of all healthy cases and can be stated mathematically as:

(3) $$\mathrm{Specificity}=\frac{TN}{TN+FP}$$

In addition to accuracy, sensitivity and specificity, Youden’s J statistic was first described in 1950 as a way to capture a single measurement performance assessment of a dichotomous diagnostic test [33]. The Youden’s J statistic is calculated as:

(4) $$J=\frac{TP}{TP+FN}+\frac{TN}{TN+FP}-1$$

Or in its simplistic form:

(5) J=Sensitivity+Specificity−1

The values of the J statistic range from 0 to 1. A test that has as zero value gives the same proportion of positive results for both those with the disease state and those without the disease state. In other words, a J value of 0 is useless in assessing the status of a patient because it provides a positive result for the same number of patients that are experiencing the disease state as those that are not. Conversely, a J value of 1 demonstrates that an assessment modality accurately identifies all subjects with a disease state and those without. Quantitative comparisons of sensitivity, specificity, and the J statistic will be used in a subsequent section of this review for comparisons between standard vital signs and hemodynamic measurements in order to identify those physiological signals required by wearable sensors to optimize diagnostic accuracy.

## 3. New Monitoring Approach: The Compensatory Reserve

### 3.1. Defining the Compensatory Reserve

**Figure 2:**
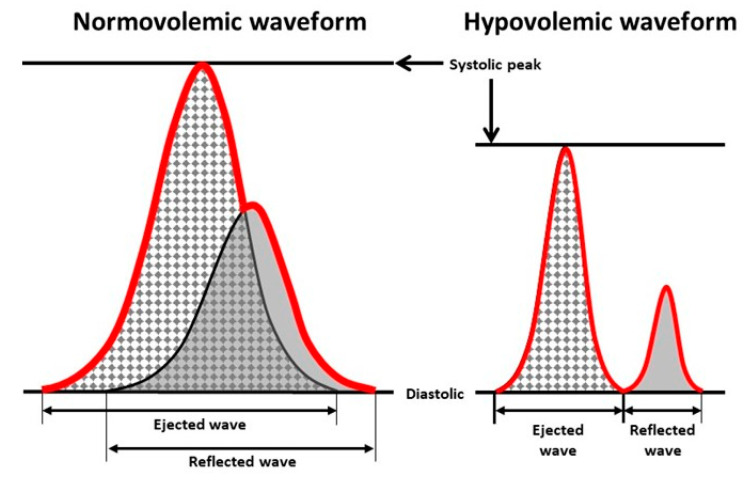
Illustration of changes in features of the ejected and reflected arterial waveforms progressing from a normal blood volume state to a state of reduced central blood volume (i.e., hypovolemia) such as that experienced during hemorrhage. The red line indicates the integrated waveform that is clinically observed. Modified from Convertino et al. [14,22,23].

**Figure 3:**
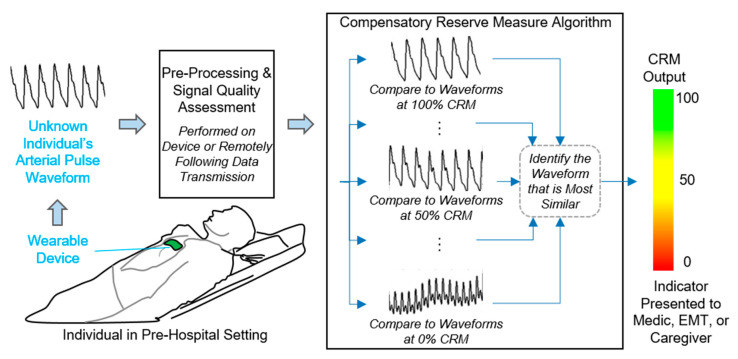
Diagram illustrating the overall framework envisioned for using the compensatory reserve measurement (CRM) in pre-hospital care, including the details on the CRM machine learning algorithm for assessing beat-to-beat analog arterial pressure waveform features in an individual patient unknown to the algorithm. The unknown arterial waveform is compared to a large waveform “library” collected from a diversity of human subjects exposed to progressive reductions in central blood volume. The algorithm identifies the most similar waveform in the waveform library with the unknown sample to generate a CRM value. Modified from Convertino et al. [14,18,21,22,23,55].

The compensatory reserve is a concept that represents the sum total of all physiological mechanisms that contribute to the maintenance of systemic DO_2_ to the body’s tissue. Conceptually, a compensatory reserve can be calculated as the difference between a baseline value at rest (100% reserve) and the value at the onset of hemodynamic instability (i.e., 0% reserve) [14,22,23,24,25,30,34,35,36]. In this regard, each individual has a finite ‘reserve’ consisting of physiological feedback mechanisms designed to compensate for low blood flow states. The complexity of this compensatory reserve is reflected by the reported observation that the physiology of integrated compensation is unique for each individual [37]. When this capacity to compensate becomes depleted, a state of decompensated shock occurs. Clinically, a compensatory reserve measurement (CRM) can be obtained from assessment of changing arterial pressure waveform morphology associated with changes in compensation [14,18,21,23,30,38,39,40,41,42,43,44,45,46,47,48,49,50,51,52,53,54]. Figure 2 illustrates that each arterial waveform consists of two primary waves: (1) an ‘ejected’ wave with features that are dictated by all compensatory mechanisms that influence myocardial function; and (2) a ‘reflective’ wave with features that are influenced by all compensatory mechanisms involved in the control of peripheral blood flow [14,22]. The LBNP model of hemorrhage has been used to generate a large reference database of more than 650,000 arterial pressure analog waveforms generated from noninvasive photoplethysmographic techniques and collected from more than 260 healthy men and women with age range of 18 to 55 years across various stages of reduced central blood volume to the point of decompensated shock (0% compensatory reserve) [22]. With application of advanced machine-learning technology to this large physiologically-diverse database, the CRM algorithm has ‘learned’ to provide rapid and continuous measures of changing arterial pressure waveform morphology to the clinical caregiver with the ability to gain an early and accurate assessment of the individual patient’s medical status without the need for demographic data or measures of the patient’s baseline physiology (as depicted in Figure 3) [21,24].

### 3.2. Performance Comparisons: Compensatory Reserve versus Vital Signs

Clinical measurements that inform and change the medical management of critically injured and sick patients should demonstrate high diagnostic accuracy. One approach to assess diagnostic accuracy includes direct comparisons of sensitivity and specificity across various monitoring capabilities. Table 2 presents such comparisons for the prediction power of standard vital signs and hemodynamic measurements for the onset of decompensated shock from data generated from LBNP experiments. The measurement of compensatory reserve displayed by far the greatest sensitivity, indicating its superior ability to correctly predict the onset of decompensated shock. Similarly, a greater specificity generated from the measurement of compensatory reserve indicated its superiority compared to the other vital signs and hemodynamic measures in the ability to identify patients who will not experience decompensated shock. The higher specificity of CRM reflects the failure of standard vital signs and hemodynamic measures alone to recognize the difference between individuals who are ‘good’ compensators from those who are ‘poor’ compensators [18,21,22,23,31,56,57,58,59]. Perhaps most striking is that standard vital signs and hemodynamic measurements have consistently been shown to lack sufficient accuracy as diagnostic tools to provide reliable clinical information [18,23,38,39,54,60,61]. In contrast, the ability of CRM to provide early reliable information with acceptable diagnostic accuracy is reflected by it being the only measurement with a Youden’s J index above the discriminative threshold value of 0.5 that confirms a useful clinical result [33,62] (Table 2).

**Figure 4:**
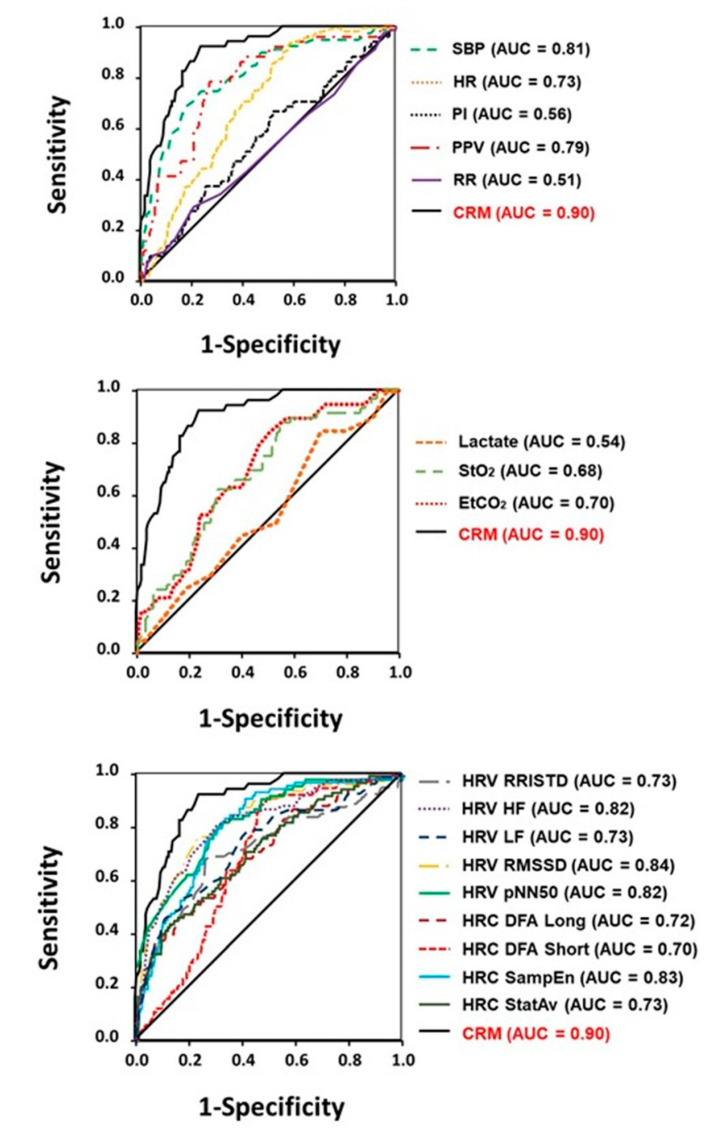
ROC AUC comparisons for prediction of onset of decompensated shock between measures of compensatory reserve (CRM) and standard vital signs (**top** panel), metabolic metrics (**middle** panel), and autonomic nervous system responses measured by indices of heart rate variability (HRV) and complexity (HRC) (**bottom** panel). SBP, systolic blood pressure; HR, heart rate; PI, perfusion index; PPV, pulse pressure variability; RR, respiratory rate; StO_2_, tissue oxygen saturation; EtCO_2_, end-tidal carbon dioxide; RRISD, R-to-R interval standard deviation; HF, high frequency; LF, low frequency; RMSSD, root mean square standard deviation; pNN50, percentage of RRI that vary by at least 50 ms; DFA detrended fluctuation analysis; SampEn, sample entropy; StatAv, stationarity.

The performance of standard vital signs and hemodynamic measurements to provide an early and accurate prediction for onset of decompensated shock can also be assessed with comparisons of sensitivity and specificity calculated using the Area Under the Curve (AUC) Receiver Operating Characteristic (ROC) statistical analysis. Figure 4 provides ROC AUC comparisons of CRM with various hemodynamic (top panel), metabolic (middle panel), and autonomic cardiac (bottom panel as represented by metrics of heart rate variability and complexity) responses. The ROC AUC data in Figure 4 are based on human data generated from experimentally-controlled progressive reductions in central blood volume using the LBNP hemorrhage model [38,39,54,56,57,63]. Similar results have been reported from experiments involving controlled hemorrhage in humans [25,61,64]. These latter data corroborate the results presented in Table 2 that arterial waveform feature analysis provides a monitoring technology with the greatest ability for early and accurate prediction for the onset of decompensated shock.

Optimal management of significant traumatic hemorrhage and other compromising clinical conditions is often delayed by failure to recognize a medical crisis due to the current reliance on traditional vital signs and/or other standard physiological measures that represent a limited assessment of a totally integrated compensatory response [22,24,25,26,27,28,29,54,61,64]. In this regard, the value of monitoring the arterial waveform morphology for early detection of a clinical crisis using a CRM algorithm has been well documented during actual controlled human hemorrhage in the laboratory setting [14,22,25,38,39,40,41,42,44,50,52,53,61,64,65], and translated to early recognition of hypovolemia and hypotension when used by first responders during simulated emergencies training exercises [66,67], and in hospital critical care settings [20,21,43,45,46,47,49,51,60,68,69,70,71,72]. The comparative data regarding sensitivity, specificity and diagnostic accuracy of various monitoring technologies presented in this review provide compelling support for the notion that the development of wearable sensors must include an ability to capture analog signals that allow for continuous real-time analysis of changes in features of the analog arterial waveform. It should be recognized that a functional FDA-cleared monitoring system with the CRM algorithm integrated into a standard finger pulse oximeter has been developed and tested [20,22,30,69]. However, such technology has proven to provide limited information to the clinical caregiver about patient status because of unstable positioning and movement artifact. In this regard, we use the following sections of this review to emphasize the need for developing new wearable sensor technologies that can be integrated with the established CRM algorithm in order to advance vital sign monitoring for emergency critical care.

## 4. Current Sensor Technology

### 4.1. Arterial Waveform Measurement Modalities Amenable to Wearable Technology: Obtaining Reliable High Signal-to-Noise Features

The original studies establishing the basis for CRM [30] used arterial waveforms measured by volume-clamping based continuous blood pressure measurement technology (i.e., Finapres) [73]. Such arterial waveforms have been demonstrated to accurately represent corresponding peripheral blood pressure waveforms obtained using arterial lines [74], and are thereby considered to be a reference standard for non-invasive continuous blood pressure measurement. While the volume-clamping technique is quite accurate at acquiring analog arterial pressure waveforms, the system required is expensive, large, and power-hungry, and thus unsuitable for point-of-care settings. Accordingly, to facilitate translation of CRM outside the lab, investigators have explored other techniques for obtaining analog arterial waveforms that *resemble* blood pressure waveforms; namely, the most commonly employed signal has been the photoplethysmogram (PPG) [39].

**Figure 5:**
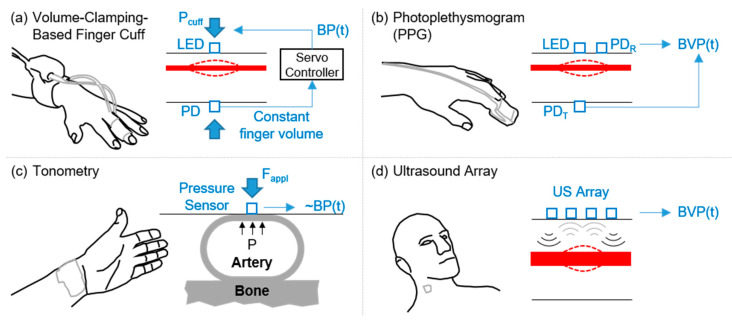
Illustration of different waveforms capture techniques that can provide a blood pressure (BP(t)) or blood volume pulse (BVP(t)) signal. (**a**) Volume-clamping based finger-cuff BP measurement (i.e., Finapres). (**b**) Photoplethysmography (PPG) based blood volume pulse measurement. (**c**) Tonometry based arterial pulse measurement. Image created based on Lee and Nam [75]. (**d**) Ultrasound array based arterial pulse measurement. Image created based on Wang and Xu [76].

Example sensing modalities that provide arterial pressure waveforms (or analogs) that are directly amenable to CRM are summarized in Figure 5. Figure 5a shows volume-clamping based finger cuff BP measurement (i.e., Finapres), which uses an LED and photodiode (PD) to capture the blood volume as a function of time in the finger, and uses a servo controller to modify cuff pressure (P_cuff_) dynamically to set the finger blood volume to a constant level. The output pressure required from the controller is thus the hemodynamic pressure inside the artery, and a waveform can be outputted representing the continuous arterial BP signal. The measurement can only be obtained from the finger.

Note that the approaches besides volume-clamping based BP measurement would result in waveform characteristics that would differ from the existing library of LBNP based arterial waveform measures used for CRM. Thus, a small data collection of approximately ten subjects may be needed with the new modality such that transfer learning or fine tuning methods for retraining the algorithm can be implemented. Following such methodology, the existing database can still be leveraged with the new sensing modality to yield accurate CRM results.

#### 4.1.1. PPG Signals

Figure 5b shows PPG measurement, which hinges on the acquisition of the BVP waveform, by illuminating a tissue volume with an LED and measuring the transmitted light through the tissue with a PD (PDT) or the light reflected back from the tissue volume with a PD (PDR) [53,77]. The measurement is most commonly obtained from the finger in transmissive mode, but in reflective mode can be measured from other well perfused sites on the body (e.g., forehead, forearm, wrist). PPG is the basis for pulse oximeters, used ubiquitously for measuring arterial oxygen saturation in clinical settings. With each heartbeat, the volume of arterial blood in the tissue being illuminated decreases during diastole and increases during systole, and thus the light passing through the tissue is brighter (diastole) and dimmer (systole) at the photodiode. Since the volumetric expansion and contraction of the arteries is dependent on pulse pressure and arterial compliance, the PPG waveform closely resembles the underlying arterial blood pressure waveform in shape. While PPG waveforms are captured on commercially available pulse oximeter instrumentation, such waveforms may not be reliable for CRM since the PPG signals are heavily filtered and processed [77]. PPG can be measured in both transmissive and reflectance mode: for transmissive mode operation, the LED and photodiode are on opposite sides of the tissue (typically the earlobe, fingertip, or toe); for reflectance mode operation, the LED and photodiode are adjacent to one another on the same side of the tissue, and thus the locations for measurement can theoretically be anywhere on the body with sufficient perfusion (e.g., forehead, forearm, chest, and wrist). The main disadvantages of reflectance-mode PPG are that the signal quality is lower [78], the measurement varies with positioning and the distance between the LED and the photodiode, and the signal is more affected by motion artifacts [79,80]. Recent developments in device fabrication have allowed PPG sensing systems to be flexible and skin-interfaced for comfortable use in long-term care scenarios [81,82]. Soft and stretchable optoelectronics sensing for transmissive PPG measurement was demonstrated by Biswas, et al. [83]. An interesting approach not requiring an LED but rather using ambient light for PPG sensing was demonstrated by Han, et al.; with this approach, PPG signals with distinguishable heartbeat peaks were recorded and corresponding pulse oximetry readings were obtained [84].

#### 4.1.2. Tonometry Signals

Figure 5c shows tonometry measurement, which involves the application of a force to flatten (or applanate) an artery with a given applanation force (F_appl_), and a pressure sensor applied to the skin above the artery then records the time varying fluctuations in pressure applied by the blood on the arterial wall [85,86]. With perfect applanation, this pressure waveform (BP(t)) is exactly equal to arterial pressure; however, in practice, applanation is usually imperfect and thus the waveform simply resembles BP. The most common measurement site is the radial artery. The advantage in tonometer measurements as compared to PPG is that substantially lower power consumption is required [87]—PPG employs active sensing where light is delivered to the tissue and then the resultant transmitted or reflected light level is detected; tonometry is a passive measurement where a transducer simply records the distension of the arterial wall. However, the major disadvantage in tonometry as compared to PPG is that the measurement is highly dependent on the location, and the transducer must be reliably placed over a superficial artery. 

#### 4.1.3. Wearable Ultrasound

Figure 5d shows ultrasound array based measurement, which uses an array of ultrasound transducers in a flexible form factor placed on the skin to measure arterial diameter changes versus time for a large artery (e.g., the carotid artery). Changes in arterial diameter correspond to the BVP, but are measured from a deeper artery as compared to PPG or tonometry, and thus may be less affected by vasoconstriction. A common measurement site is the carotid artery. Recent work has demonstrated that a blood pressure waveform can be measured from the surface of the skin based on this principle using a nano-engineered flexible ultrasound array [88]. The device acquires time-varying changes in blood vessel diameter, which are then mapped to an estimate of the underlying blood pressure waveforms. By employing ultrasound to measure this pulsating blood vessel diameter, the device can focus on larger arteries, namely the carotid, which are deeper under the skin than PPG- or tonometer-based approaches can access. Accordingly, reduced sensitivity to sensor positioning has been demonstrated as compared to tonometry, and accurate extraction of arterial pressure waveforms has been achieved [88]. Note that this approach requires calibration to acquire the absolute blood pressure values (i.e., systolic, diastolic, and mean arterial pressure), but the waveforms measured are likely the closest to the underlying blood pressure waveforms of the three prior modalities discussed here. An additional concern that should be noted with this approach is that the detection of the artery may require manual positioning and/or image annotation in broad use; however, the array of transducers employed on the device may limit such a need for expert annotation. 

#### 4.1.4. Cardio-Mechanical Vibrations

While CRM to date has focused on arterial pulse waveforms measured peripherally, there have also been studies employing cardiogenic vibration signals as an index of hypovolemia based on machine learning techniques in both human subjects (LBNP) [89] and animal models [90]. Note that since these measurements to do not directly yield an arterial pulse waveform, they are not depicted in Figure 5 to avoid confusion. Cardiogenic vibration signals include the seismocardiogram (SCG) and ballistocardiogram (BCG), both of which originate from the vibrations of the chest (SCG) or whole body (BCG) in response to the ejection of blood from the heart and movement of blood through the vasculature [91]. SCG and BCG signals can be measured accurately with inexpensive and commercially available sensors [92,93], and have been demonstrated to be reliable even in the presence of movement [94,95]. As with the other sensing modalities described above, soft, conformal patch based sensing of SCG signals is also possible: Liu, et al. describe an epidermal sensing system for providing mechano-acoustic measurements of cardiovascular health, including heart sounds and SCG signals [96]. Machine learning based analyses performed on these waveforms demonstrated that high quality estimation of blood volume status (analogous to CRM) could be obtained in a pig model of hemorrhage [90]. Importantly, for persons suffering polytraumatic injuries who may not have an available digit or ear, and may have extensive vascular damage that could reduce PPG waveform quality due to increased wave reflections, such cardiogenic vibrations may provide an alternative waveform for CRM-based volume status assessment.

#### 4.1.5. Other Emerging Wearable Sensing Devices

The field of wearable sensing has seen a myriad of new devices over the past several years, driven by the use of new materials and fabrication approaches, developments in chemical sensing, and the advent of soft, flexible, and stretchable electronics. These new devices promise to deliver comfortable and high-performance sensing of cardiovascular health parameters with thin, flexible, and stretchable mechanical footprints that resemble the properties of human skin. Emerging technologies of interest also include biodegradable sensors such as the one described in Boutry, et al., for tonometry-based pulse signature sensing [97], and combined chemical/electrophysiological hybrid biosensing systems such as the one presented in Imani, et al. [98]. Additionally, while not discussed in detail here, wearable sensing systems measuring impedance plethysmogram waveforms [99], or magnetic inductance based cardiac waveforms [100], may also be employed.

Table 3 provides a comparison of state-of-the-art sensing technologies for arterial pulse waveform analogs, including summarizing the principle of operation, the typical locations on the body where the signals are captured, and the advantages and disadvantages of each method for application to CRM. 

### 4.2. Mitigating the Effects of External Vibrations and Motion Artifacts

**Figure 6:**
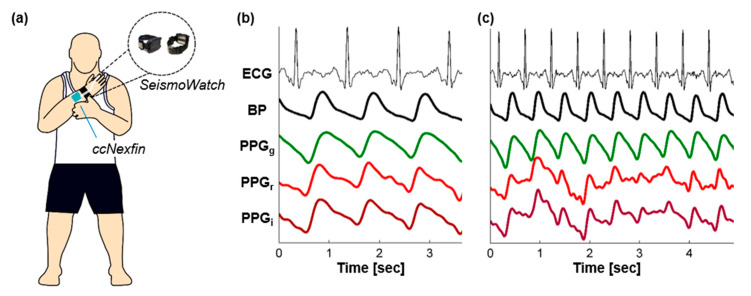
Example PPG signals taken together with electrocardiogram (ECG) and BP measurements as a reference for comparison. (**a**) The signals were obtained by the Georgia Tech SeismoWatch hardware as described in Ganti, et al. [101]. The BP waveforms shown for comparison were obtained with the ccNexfin volume-clamping based finger cuff BP system (Edwards Lifesciences). (**b**) Signals measured from a subject at rest. Note that the PPG waveforms closely resemble the BP waveforms in shape, with the red and IR (PPG_r_ and PPG_i_, respectively) containing many of the same characteristics expected in an arterial pulse waveform, while green (PPG_g_) appears to be a smoothed version of the BP waveform. (**c**) Signals measured from the same subject following heavy exercise with motion artifacts corrupting the waveforms. The red and IR signals are corrupted heavily while the green PPG signal quality remains high.

In point-of-care settings, sensing systems for obtaining arterial pulse waveforms often encounter external vibration or motion related artifacts (e.g., battlefield settings or civilian patient transport). These artifacts can greatly impact the quality of the waveforms that are measured, and result in errors in the computation of clinically relevant information (e.g., CRM). External vibrations from transport vehicles during en route care, for example, can be quite large (e.g., on an ambulance or helicopter). Motion artifacts will always be present in the measured signals, unless the patient is unconscious, and may result from either whole-body movements or even more subtle sources such as respiration, talking, or coughing. There are many approaches for mitigating the effects of vibration and motion artifacts in arterial waveform measurements, but the most common techniques involve: (1) improving the signal quality at the source as much as possible, (2) providing auxiliary sensors to detect and cancel motion artifacts from the measured waveforms, and (3) quantifying signal quality on a beat-by-beat basis to facilitate rejection of lower quality waveforms from the subsequent data analysis. Figure 6 shows an example of BP and PPG signals (green, red, and infrared wavelength) captured from a representative subject using a wearable watch technology [101] (a) while at rest (b) and following vigorous exercise with example motion artifacts (c). 

#### 4.2.1. Improving the Signal Quality at the Source

For reflectance-mode PPG signals, signal quality is optimized at the source against motion artifacts through the use of green wavelengths rather than red or infrared (IR) [79,102,103]; green penetrates less deeply into the skin, and thus is less attenuated through the forward and backward path through the tissue. Providing non-zero contact pressure between the PPG sensor and the skin can also increase the amplitude of the measured waveforms [53,104]. Specifically, the PPG amplitude is maximized when the contact pressure is equal to the mean arterial pressure (i.e., the transmural pressure is zero). Thus, to reduce the impact of motion artifacts, green wavelengths can be employed for PPG detection, and a non-zero contact pressure can be applied between the sensor and the skin to optimize signal level. The waveforms shown in Figure 6c visually demonstrate this relationship between wavelength and resultant PPG signal quality during motion artifacts. While the red and IR PPG signals are quite heavily affected by the motion artifacts, the green PPG signal quality remains high. Nevertheless, note that many of the key waveform features captured by the red and IR PPG are missing in the green PPG signal due to the fact that the green signal captures primarily the superficial cutaneous vasculature while red and IR penetrate deeper into the skin.

For tonometry-based arterial pulse waveform measurements, optimizing signal quality at the source fundamentally requires robust coupling between the superficial artery and the sensor. Tonometry requires a backing force such that the sensor remains consistently in contact with the arterial wall throughout the measurement duration. Thus, a strap is typically used to provide such backing force, for example for radial artery tonometry, and the tightness of the strap must be optimized to be high enough such that the sensor remains in contact with the artery but not high enough to occlude the artery [105]. To reduce the variability due to sensor placement, arrayed sensors are also often used for tonometry based recordings [106]. The sensing system can thus be placed over the palmar aspect of the wrist near the radius bone, and software based approaches can be used to find the waveform with the highest signal quality from the array of sensors. 

Techniques for optimizing wearable ultrasound array based arterial pulse waveforms are not yet well understood since the measurement modality is relatively new. However, from an intuitive perspective it is likely that the ability to accurately place the ultrasound array in the proximity of the artery from which measurements will be taken (e.g., the carotid artery), and the coupling between the sensor array and the skin (likely requiring acoustic matching such as ultrasound gel), will play important roles in ensuring high quality waveforms are obtained. 

For cardiogenic vibration signals, there are also several aspects that must be considered to optimize signal quality at the source. First, the sensing system should use a sensor with sufficiently low noise floor to capture the micro-vibrations. In the case of SCG signals for example, only accelerometers with input-referred noise of 50 µg_rms_/√Hz or lower should be used. The standard accelerometers deployed on wearable sensing systems and smartphones for inertial measurement have much higher noise than this, with values typically in the 150–300 µg_rms_/√Hz range. Second, leveraging the information from all three axes of the SCG signal, or even including rotational components (i.e., gyrocardiography) as captured with a gyroscope, has been demonstrated to yield greater information than the dorso-ventral axis alone [107]. Third, and perhaps most importantly, the sensor should be rigidly adhered to the body such that movement of the person wearing the sensor does not lead to detachment or other major mechanical disturbances.

#### 4.2.2. Providing Auxiliary Sensors to Detect and Cancel Motion Artifacts

A commonly-used technique for reducing the impact of motion artifacts on PPG signals is the inclusion of an auxiliary accelerometer to detect and provide digital subtraction of motion artifacts [108,109,110]. The captured acceleration signal provides a noise reference that can be used via adaptive noise cancellation or other signal processing approaches to remove such artifacts from the PPG signal. An alternative approach to reducing motion artifact influence on wearable cardio-mechanical signals leverages auxiliary sensing to capture other signals of cardiovascular origin, namely the electrocardiogram (ECG) [111,112]. Subsequently, rather than removing motion artifacts, the signal strength itself can be bolstered. While the authors are not aware of such auxiliary sensor-based methods for increasing robustness to motion artifacts in tonometry and ultrasound-based arterial waveform capture modalities, intuitively such methods should be directly applicable to these modalities as well. The fundamental approach of either providing a noise reference for noise cancellation or a timing reference for ensemble averaging or otherwise strengthening the signal characteristics are valid for these modalities similarly as for PPG signals. 

For cardiogenic vibration waveforms, several approaches have been demonstrated in the existing literature for detecting and cancelling artifacts due to motion or external vibration. Auxiliary sensors for detecting or cancelling motion artifacts from BCG signals include foot electromyogram (EMG) sensing to determine periods of elevated motion as well as external geophone based recordings of floor vibrations for subsequent cancellation [113,114]. Furthermore, signal enhancement using concurrent ECG signals for ensemble averaging, synchronized moving averaging, and otherwise beat segmentation is standard practice.

#### 4.2.3. Quantifying Signal Quality for Rejecting Lower Quality Waveforms

A third approach that can be leveraged to mitigate the effects of external vibrations and motion artifacts on arterial pulse waveforms is the automatic quantification of signal quality on a beat-by-beat basis. Such signal quality assessment is an important tool towards quantifying when the waveforms should be inputted to subsequent machine learning steps (e.g., CRM computation) or, alternatively, when waveform segments should be rejected. Signal quality indices have thus been developed for PPG and cardio-mechanical signals, and have been validated in recent literature [115,116,117]. The challenge in such algorithms is that both the signal (of cardiac origin) and the noise are non-stationary, and there is substantial variability in signal shape across subjects and also sensor locations. Thus, conventional approaches such as matching the morphology of measured PPG (or tonometry, ultrasound, cardiogenic vibration signals, etc.) to previous recordings or a database of recordings is not an appropriate technique. Rather, waveform matching must be accomplished using techniques such as dynamic time warping (DTW) [118], which allow for stretching of each beat against the templates with which the beat is compared. DTW-based approaches have demonstrated promise for arterial pulse signals [116]. The establishment of such automated techniques for signal quality assessment—as compared to manual annotation which has been employed in many studies in the existing literature—will represent an important step towards facilitating translation of these sensing approaches to point-of-care settings. Note that, whenever possible, techniques for improving signal quality should be employed rather than techniques for only assessing signal quality. However, in practical settings, many sources of artifacts, noise, and interference corrupting physiological measurements cannot be completely attenuated by signal capture optimization, nor can they be completely removed by auxiliary sensors and associated noise reduction algorithms; thus, the ability to detect and remove low quality events is a key element in delivering robust and reliable CRM outputs to caregivers for subsequent clinical decision making. 

### 4.3. Eliminating the Need for Baseline Measures/Calibration

The use of wearable sensors for CRM-based hypovolemia assessment in field settings may not allow for baseline data to be obtained; for example, if one envisions a person injured in a major car accident, an emergency medical technician (EMT) may simply apply a wearable patch or system to the person when arriving on the scene after exsanguination has begun. Accordingly, algorithms for quantifying compensatory reserve based on machine learning should be globalized rather than designed in a patient-specific manner (see Figure 3). Features leveraged by the algorithm should thus be based on relative measures (e.g., timing intervals, variability measures, etc.) rather than absolute measures (e.g., absolute amplitude of the signal). Moreover, machine learning algorithms should be trained using leave-one-subject-out cross-validation (LOSO-CV) rather than *n*-fold CV, with at least one subject deliberately left out of the training set such that the algorithm focuses on global trends in the sensed waveforms. Finally, since sensor placement can impact the shape of waveforms measured for many reflective PPG [79], tonometry, ultrasound-based blood pressure, and cardiogenic vibration signals [117], such placement-dependent changes should be thoroughly quantified, and methods for harnessing underlying dynamics should be leveraged as compared to features that require manual annotation [118].

### 4.4. Real-Time Measurements and Processing for Display

An important consideration is how to display the resultant information derived from the arterial waveforms to the physician or caregiver. One option is to provide a dashboard type display with perhaps a single numerical value indicating the compensatory status of the person (i.e., a CRM value). Another option might be a red, yellow, or green indicator to provide information regarding the clinical decisions to be made during triage (Figure 3). An exciting opportunity exists in the pairing of the volume status information delivered through the automatic analysis of the arterial pulse waveforms with autonomous critical care systems for combat casualty care. Scientists in the academic and commercial domain are conducting research designed to develop systems and methods for providing fluids autonomously to combat casualties based on physiological data [119,120,121,122]; providing more in-depth measurements of volume status beyond traditional vital signs to such systems may yield improved results in managing fluid for hemorrhaging patients or casualties. As different applications and use cases emerge, it will be important to determine what processing will be applied at what stage in the system. For example, in one implementation the signals may be wirelessly transmitted from the wearable sensing system to a local smartphone, tablet, laptop, or other dedicated receiver, at which point algorithms may be implemented on that receiver device to output a CRM to be displayed to the caregiver. Another implementation that is possible is to incorporate the CRM machine learning algorithm into the wearable hardware itself (i.e., computing on the edge), in which case the CRM value itself may be transmitted wirelessly or a readout may be provided on the hardware itself. Regardless of where in the signal chain the processing is implemented, it will be necessary to consider security and privacy concerns of the patient, as well as power consumption and associated battery life on the wearable hardware itself. 

### 4.5. Electronic Documentation in the Prehospital Setting

The ability to collect and analyze large quantities of data from trauma patients, particularly in austere prehospital settings such as the battlefield, hinder the potential for understanding and improving clinical process and performance [123]. In situations where battery life must be extended for as long as possible, or when wireless transmission is otherwise not feasible, data storage locally on the sensing system may be desired and implemented using micro secure digital (microSD) cards or other non-volatile memory on board the system. The advantage to such local storage of all physiological waveforms is that a detailed record can be kept of the data for subsequent analysis and/or evaluation of the treatment approaches employed. Data extracted from all patients could then be used to retrospectively determine which approaches were most successful, and care can then be optimized accordingly with this evidence. In some instances, the amount of data being stored may be quite large, and may necessitate compressed sensing approaches prior to digitization [124,125]. However, in most cases—since physiological signals such as the PPG are typically of low bandwidth (<100 Hz)—direct digitization and storage of data are feasible for many weeks of continuous recording.

### 4.6. Military Perspectives and Implications

In December 2013, the Director of the former Directorate of Combat and Doctrine Development (currently the Capability Development Integration Directorate) signed a ‘Requirements Adjudication Team’ memorandum that documented a military medical requirement for the measurement of compensatory reserve. The Committee on Tactical Combat Casualty Care reaffirmed this requirement by recommending “continued development and expedited fielding of technologies (such as the compensatory reserve) that enable prehospital combat medical personnel to better evaluate the need for and the adequacy of fluid resuscitation” [126]. As military missions of the future will be performed in complex multi-domain operations (MDO) and/or involve large scale combat operations (LSCO) with a possibility of limited air superiority, delays in early and rapid medical evacuation in addition to mass casualty scenarios will require individualized triage decision support that will prove critical for successful execution of prolonged field care. In the military setting, warfighters could wear a sensor embedded on a wrist watch (e.g., Figure 6) or as part of their fighting ensemble system so that the clinical status of injured casualties could support continuous hands-free documentation by a combat medic using a remote monitoring device (e.g., phone). Since previous research has also identified the CRM with the capability to track physical and physiological performance [55,70,127], a military wearable sensor system that integrates the continuous monitoring of CRM could be used by unit commanders as a real-time metric of performance readiness (e.g., manage impact of heat strain and/or dehydration) as well as its use for optimizing combat casualty care of warfighters in austere battlefield settings. 

### 4.7. Future Directions

Future work is required to collectively advance the vision of enabling CRM-based assessment of hypovolemia in field settings. Wearable sensing systems are needed with minimally obtrusive form factors facilitating the accurate measurement of arterial pulse or cardio-mechanical waveforms outside of laboratory settings. Such systems should likely employ multi-modal sensing approaches: for example, PPG sensing can be combined with tonometry and/or cardiogenic vibration sensing to ensure that if one modality experiences artifacts from motion or other confounding variables, the other modality might still accurately capture cardiac signatures. The physiological origins of the signals being measured, and the manner in which confounding variables such as environmental factors (e.g., ambient temperature), arrhythmias, other cardiovascular disease conditions, and high body mass index of the patient may impact the algorithms and/or sensor design should be investigated further. The specific features and signal modalities that might offer the most salient information regarding volume status should continue to be studied through LBNP, heat stress/dehydration, and other hypovolemia inducing protocols. These wearable sensing systems must be paired with state-of-the-art machine learning algorithms to reduce noise and interference, automatically assess signal quality, and output a reliable and robust indication of CRM. Designers of such hardware, firmware, and software required for this framework should collaborate closely with subject matter experts such as medical professionals and EMTs such that the user interface and display offered to these professionals provides the information needed for rapid decision making in the challenging environment of prehospital trauma care. Finally, extensive validation of these technologies as a whole must be conducted to ensure that the performance of all components of the overall system are sufficiently robust to obtain regulatory approval and ultimately improve outcomes.

## 5. Conclusions

As technology advances to facilitate the emergence of autonomous medical treatment systems as well as early and accurate diagnosis and triage, the incorporation of sensors capable of supporting measurements of CRM can ensure that patients who require emergency medical care (e.g., civilian trauma patients or wounded service members) receive appropriate treatment interventions, even when medical personnel are not available. As such, the development and availability of a single advanced monitoring system that includes wearable sensors capable of capturing analog arterial waveforms and integrating them with application of machine-learning algorithms (i.e., artificial intelligence) can provide clinical and/or performance decision-support with the goal of optimizing health, safety and wellbeing in prehospital and emergency room settings. In addition to offering robust performance, human factors aspects of the sensing system design must be prioritized such that both the hardware and clinician-facing displays seamlessly integrate into the workflow, making it easier for decisions to be made in time-critical, challenging situations. Finally, such systems and associated algorithms as described in this review paper may be applied to the diagnosis or management of other cardiovascular conditions, such as heart failure management.

## Figures and Tables

**Table 1:** Qualitative timing of changes in traditional vital signs and blood chemistries during progressive central hypovolemia. Modified from Convertino et al. [14,22] and Moulton et al. [30].

Vital Sign or Measurement	Change During Progressive Central Hypovolemia
Systolic blood pressure	Late
Diastolic blood pressure	Late
Mean blood pressure	Late
Heart rate	Non-specific
Shock index (heart rate/systolic pressure)	Late
Oxygen saturation	Late
Radial pulse character assessment	Late
End-tidal CO_2_	Late, Non-specific
Respiratory rate	Late, Non-specific
Glasgow Coma Scale	Late, Non-specific
Blood pH, PCO_2_, Base Excess	Late, Non-specific
Blood Lactate	Late, Non-specific
Hematocrit, Hemoglobin	Late, Non-specific

**Table 2:** Sensitivity, specificity and Youden’s J index of traditional vital signs and hemodynamic responses for prediction of the onset of decompensated shock secondary to progressive central hypovolemia. Modified from Convertino et al. [14,22,23,25].

Vital Sign	Sensitivity	Specificity	Youden’s ‘J’ Index
Systolic Blood Pressure	0.80	0.17	0.03
Diastolic Blood Pressure	0.40	0.53	0.07
Mean Blood Pressure	0.60	0.33	0.07
Heart Rate	0.80	0.02	0.18
Stroke Volume	0.60	0.33	0.07
Cardiac Output	0.80	0.02	0.18
Pulse Pressure Variability	0.78	0.69	0.47
Peripheral Capillary Oxygen Saturation (SpO_2_)	0.60	0.00	0.40
Deep Muscle Oxygen Saturation (SmO_2_)	0.65	0.63	0.28
Compensatory Reserve	0.84–0.87	0.78–0.86	0.62–0.73

Note: For Youden’s Index, a value of 1 represents a perfect diagnostic test, while a value of 0 represents a test with poor diagnostic accuracy. Stroke volume (SV), systolic, diastolic and mean blood pressures were measured by finger photoplethysmograpy; heart rate (HR) was measured by standard electrocardiogram; cardiac output was calculated as SV times HR; Pulse pressure variability and SpO_2_ was measured with standard pulse oximetry; SmO_2_ was measured with near-infrared spectroscopy; compensatory reserve was measured by pulse oximetry.

**Table 3:** Comparison of Sensing Technologies for Arterial Pulse Waveform Analogs.

Sensing Modality	Principle of Operation	Typical Location(s)	Advantages	Disadvantages
PPG	Optical sensing of blood volume changes in a small volume of tissue	Transmissive: Finger, Earlobe, Toe Reflective: Wrist, Forehead, Forearm	Waveform resembles arterial pressure curves; signal quality is typically high; well-established sensing modality and already used in many clinical settings (i.e., pulse oximetry)	Signal originates mainly from the cutaneous vasculature and thus is affected by hypoperfusion (peripheral vasoconstriction); reflective PPG is more convenient in terms of placement but suffers from motion artifacts and placement-based variability in signal shape; requires substantial current consumption (active sensing)
Tonometry	Force/pressure sensing of arterial wall displacement at the surface of the skin	Wrist (Radial Artery)	With applanation of the artery, captures true blood pressure waveform and does not require calibration; low power measurement since it is a passive sensing approach	Applanation is challenging in practice and not reliable; measurement depends highly on placement; coupling to a superficial artery is needed
Ultrasound-Based BP	Ultrasound sensing of arterial diameter changes in deeper/larger arteries	Neck (Carotid Artery)	Measurements can be obtained from deeper arteries (e.g., carotid) and thus are less affected by hypoperfusion and/or vasoconstriction; arrayed sensing approach may reduce the variability in signal shape due to sensor positioning	Active measurement which requires substantial power consumption to deliver ultrasound energy to the body and process the resultant signals; may require manual approaches to annotating images
Cardiogenic Vibration	Mechanical vibration sensing of blood movement through vasculature	Chest (Sternum)	Passive measurements that can be captured non-intrusively with sensors on the chest; represent more central cardiac activity since the origination is from cardiac vibrations rather than peripheral blood volume pulse; minimal affects due to peripheral vasoconstriction	Not a direct arterial pressure waveform analog; requires coupling to the chest with an adhesive; may be sensitive to positioning of the sensor on the body

## Copyright

V.A.C., S.G.S., E.K.W., S.C., M.E.S., and M.J.T. contributed to this manuscript as part of their official duties for the federal government. Therefore, copyright is not applicable.
